# Excretion patterns of coccidian oocysts and nematode eggs during the reproductive season in Northern Bald Ibis (*Geronticus eremita*)

**DOI:** 10.1007/s10336-015-1317-z

**Published:** 2016-02-04

**Authors:** Didone Frigerio, Lara Cibulski, Sonja C. Ludwig, Irene Campderrich, Kurt Kotrschal, Claudia A. F. Wascher

**Affiliations:** 1 Core Facility Konrad Lorenz Forschungsstelle for Behaviour and Cognition, University of Vienna, Fischerau 11, 4645 Grünau im Almtal, Austria; 2 Department of Behavioural Biology, University of Vienna, Althanstrasse 14, 1090 Vienna, Austria; 3 Game & Wildlife Conservation Trust, The Coach House, Eggleston Hall, Barnard Castle, DG12 0AG UK; 4 Department of Animal Production, Neiker-Tecnalia, Vitoria-Gasteiz, Spain; 5 Animal and Environment Research Group, Department of Life Sciences, Anglia Ruskin University, Cambridge, UK

**Keywords:** Parasite burden, Northern Bald Ibis, *Geronticus eremita*, Reproduction

## Abstract

Individual reproductive success largely depends on the ability to optimize behaviour, immune function and the physiological stress response. We have investigated correlations between behaviour, faecal steroid metabolites, immune parameters, parasite excretion patterns and reproductive output in a critically endangered avian species, the Northern Bald Ibis (*Geronticus eremita*). In particular, we related haematocrit, heterophil/lymphocyte ratio, excreted immune-reactive corticosterone metabolites and social behaviour with parasite excretion and two individual fitness parameters, namely, number of eggs laid and number of fledglings. We found that the frequency of excretion of parasites’ oocysts and eggs tended to increase with ambient temperature. Paired individuals excreted significantly more samples containing nematode eggs than unpaired ones. The excretion of nematode eggs was also significantly more frequent in females than in males. Individuals with a high proportion of droppings containing coccidian oocysts were more often preened by their partners than individuals with lower excretion rates. We observed that the more eggs an individual incubated and the fewer offspring fledged, the higher the rates of excreted samples containing coccidian oocysts. Our results confirm that social behaviour, physiology and parasite burden are linked in a complex and context-dependent manner. They also contribute background information supporting future conservation programmes dealing with this critically endangered species.

## Zusammenfassung

**Ausscheidungsmuster von Kokzidienoozysten und Nematodeneiern während der Fortpflanzungsperiode bei Waldrappen (*Geronticus eremita*)**

Individueller Fortpflanzungserfolg hängt weitgehend von der Fähigkeit ab, Verhalten, Immunfunktion und die physiologische Reaktion auf Stress zu optimieren. In der vorliegenden Studie untersuchten wir Zusammenhänge zwischen Verhalten, fäkalen Steroidmetaboliten, hämatologischen Parametern, Parasitenausscheidungsmustern und Fortpflanzungserfolg in einer vom Aussterben bedrohten Vogelart, dem Waldrapp (*Geronticus eremita*). Insbesondere untersuchten wir Zusammenhänge zwischen ausgeschiedenen immunoreaktiven Corticosteronmetaboliten, dem Verhältnis von Heterophilen zu Lymphozyten, Hämatokrit und dem Sozialverhalten mit der Menge ausgeschiedener Parasiten und mit Fitness-Parametern, wie der Anzahl der gelegten Eier und der flügge gewordenen Jungvögel. Die Anzahl der Kotausscheidungen, die Parasiteneier enthalten, steigt tendenziell mit der Umgebungstemperatur. Verpaarte Vögel schieden deutlich öfter Kot mit Nematodeneiern aus, als Unverpaarte. Zudem enthielt der Kot der Weibchen signifikant öfter Nematodeneier, als der der Männchen. Individuen mit einem hohen Anteil an Kokzidienoozysten im Kot wurden häufiger von ihren Partnern gekrault, als Individuen mit geringeren Ausscheidungsraten. Je mehr Eier ein Individuum während der Brutzeit ausbrütete, und je weniger Nachwuchs flügge wurde, desto höher war die Rate von mit Kokzidienoozysten befallenen Proben. Unsere Ergebnisse bestätigen, dass Sozialverhalten, Physiologie und Parasitenbelastung in einer komplexen Weise miteinander verbunden sind. Darüber hinaus unterstützt die vorliegende Studie das Management dieser vom Aussterben bedrohten Vogelart.

## Introduction

Individual lifetime reproductive success largely depends on the ability to optimize the trade-off relationships between behaviour, immune function and other physiological parameters over the life history of that individual (Sheldon and Verhulst 1996; Barnard et al. 1998). For example, social context is among the most potent stressors in group-living individuals (von Holst 1998; Klein and Nelson 1999; de Vries et al. 2003; Wascher et al. 2008a, b). Activation of both the acute and chronic physiological stress responses is adaptive in nature, contingent on specific ecological and habitat pressures (Wingfield et al. 1998; Boonstra 2013). Chronic stress may even have pathological consequences (Råberg et al. 1998; Bartolomucci et al. 2005), mainly by metabolic overstimulation and by negative effects on the immune system (Azpiroz et al. 2003; Berger et al. 2005; Graham et al. 2012). The physiological stress response also appears to link immune functions with parasite burden. Both of the latter are known to co-vary with season (Bakuza and Nkwengulila 2009), sex (Zuk and McKean 1996; Tschirren et al. 2003), age (Hayward et al. 2009), personality (Barber and Dingemanse 2010; Koprivnikar et al. 2012), hierarchy (Zuk et al. 1997; Ungerfeld and Correa 2007; Muehlenbein and Watts 2010), crowding (Raouf et al. 2006) and reproductive effort (Klein and Nelson 1999).

The reproductive season is stressful in terms of social behaviour and costly in terms of energetic input (e.g. Kotrschal et al. 1998). In male chamois (*Rupicapra rupicapra*) faecal androgen and cortisol metabolites as well as parasite levels increase during mating (Corlatti et al. 2012); in Southern rockhopper penguins (*Eudyptes chrysocome chrysocome*) leucocyte profiles (i.e. the variation in granulocyte/lymphocyte ratios) but not individual body condition vary according to sex and breeding stage (Dehnhard et al. 2011). Parental care in birds is also modulated by parasite load of the offspring. In Blue tits (*Parus caeruleus*), parents increase feeding activity to compensate for the detrimental effects of ectoparasitism on the offspring (Tripet and Richner 1997), whereas male Spotless starlings (*Sturnus unicolor)* reduce their effort when caring for parasitized offspring (Avilés et al. 2009).

Overall, it would appear that reproductive success is negatively related to both parasite burden (Marzal et al. 2005; Hillegass et al. 2010; Gooderham and Schulte-Hostedde 2011) and to the activity of the immune system (Moreno et al. 1999; Møller et al. 2003; Bowers et al. 2012). A meta-analysis of 25 bird species revealed that clutch size generally increases with anti-parasite response (Martin et al. 2001; Vinkler and Albrecht 2011). In the long run, high parasite burden is linked to poor body condition (Pap et al. 2013) and increased mortality (Rousset et al. 1996; Cooper et al. 2012; Holand et al. 2014).

Haematological parameters are representative indicators of health, body condition and/or stress (Gavett and Wakeley 1986; Vinkler et al. 2010). For example, haematocrit (HCT) is considered to be an indicator of general condition in wild birds and is modulated by a number of different factors, such as age, sex, geographical elevation, energy expenditure, parasitism, nutrition and genetics (Verhulst et al. 2002; Fair et al. 2007). The heterophil/lymphocyte ratio (H/L ratio) accurately represents the general response to stressful conditions, increasing within a few hours following a stressor (Maxwell 1993; Vleck et al. 2000; Davis et al. 2008; Lebigre et al. 2012).

In the present study, we investigated environmental (temperature), individual (sex, age, number of eggs laid and fledglings) and social (pairbond status, affiliative behaviour and agonistic interactions) factors affecting parasite burden (excreted coccidian oocysts and nematode eggs) as well as different physiological parameters (HCT, H/L ratio, excreted immune-reactive corticosterone metabolites) and their possible correlation with individual reproductive output. The study was conducted in 2 years (the springs of 2010 and 2012) in a free-roaming and individually marked colony of the critically endangered Northern Bald Ibis (*Geronticus eremita*) at the Konrad Lorenz Research Station (Austria).

We hypothesized that social context affects the physiology of Northern Bald Ibises. In order to test this hypothesis we investigated the impact of several social factors (e.g. pairbond status, frequency of social interactions) on an individual’s physiology. Because physiology is linked with immune function, we predicted that social factors would also correlate with parasite burden. For example, parasite excretion may be lowered by the stress-reducing effect of social support via the presence of a pair-partner (von Holst 1998; de Vries et al. 2003; Scheiber et al. 2005; Wascher et al. 2009). However, it can also not be excluded that social interactions may facilitate parasite transmission (e.g. birds: Brown and Brown 1986; Fecchio et al. 2011; mammals: Hillegass et al. 2008; MacIntosch et al. 2012). Accordingly, individuals involved in less agonistic but more affiliative interactions than others are expected to excrete comparatively lower levels of corticosterone immune-reactive metabolites of corticosterone (BM) (Kime 1995) as well as relatively fewer samples containing nematode eggs and coccidian oocysts. The opposite would apply for individuals involved in more agonistic and less affiliative behaviour.

In many avian species breeding success has been demonstrated to be age-specific, increasing through the early years of breeding, then undergoing little change till late in life (Mauck et al. 2004) when senescence starts decreasing reproductive capabilities (Vleck et al. 2007). In the context of our study we expected reproductive success to be positively correlated with age, as none of the focal birds was very old and most of the subjects were at an early stage of their reproductive life (Table 1). However, individuals investing in reproduction may pay a (high) cost in terms of immunity (allocation trade-off) and thus exhibit a poor immune status. Northern Bald Ibis males and females contribute relatively equally to parental care (Pegoraro and Thaler 1985; Pegoraro 1992), and androgen levels have been shown to be similar between the sexes, even during the mating period (Sorato and Kotrschal 2006). Hence, we did not expect sex differences in BM; however, other factors, such as sex differences in metabolic rates, may have the potential to affect parasite burden and haematological parameters. For these reasons Northern Bald Ibis is an ideal model bird species to study relationships between physiological parameters, parasite burden and reproductive output.

## Materials and methods

### Study area and population

A free-ranging colony of Northern Bald Ibis was established in 1997 at the Konrad Lorenz Research Station (KLF; Grünau im Almtal, Upper Austria; 47°48′E, 13°56′N) by hand-raising zoo-bred chicks (Tuckova et al. 1998; Kotrschal 1999, 2003, 2007; Szipl et al. 2014) in coordination with the European Breeding Programme (Böhm 1999). The KLF flock has been raising chicks autonomously since 2000, and the colony has grown to more than 40 individually marked birds. The flock is housed and bred in a large aviary in the local Herzog-von-Cumberland game park. The aviary is kept open year-round, and thus the birds are free-flying and usually roam the feeding grounds in the Almtal-region, returning to their aviary for roosting at night and for breeding. Every year 15–20 chicks fledge in the colony, but many are lost, mainly to birds of prey in their first year of life (Kotrschal 2007). Predation rates in the 2 years of the study (2010 and 2012; see below for further details) are similar (KLF own unpublished records). No case of predation was recorded during both periods of data collection, and we therefore were able to exclude different predation stress conditions for the birds during the 2 years of the study (e.g. Clinchy et al. 2004; Boonstra 2013). Supplementary food is provided during winter and early spring when natural resources become limited (Böhm and Pegoraro 2011). The birds are supplied twice a day with hash made from 1-day-old chicks mixed with dry dog food. The food is dispensed at a designated feeding area inside the aviary (approx, 5 × 1.5 m) by one game keeper. The quantity of food is sufficient to feed all birds to saturation. At the time of data collection (reproductive phase) all of the birds were showing up at the feeding times. The birds are well habituated to the close presence of humans, and each bird is marked with an individual combination of colour rings. None of the birds has ever been treated against parasites. At the time of data collection in 2010 and 2012 the colony consisted of 46 and 39 individuals, respectively. During the spring of 2010 data were collected on 25 focal individuals (age range 1–13 years; mean age $\bar{\rm x }$ ± standard error (SE) = 6.69 ± 0.79 years). In the spring of 2012 data were collected on 29 focal animals (age range 1–14 years; mean age $\bar{\rm x }$ ± SE = 5.86 ± 0.75). Thirteen birds were sampled in both years. Details on the focal individuals (year of hatching, sex, year of data collection, number of eggs laid and fledglings per phase of data collection) are given in Table 1.

**Table 1:** Sex, year of hatching, year of participation in the study and number of eggs and fledglings of all focal individuals in phase 1 (2010) and phase 2 (2012) of data collection

Name	Sex^a^	Year of hatching	Year of participation	Eggs 2010 (*n*)	Fledglings 2010 (*n*)	Eggs 2012 (*n*)	Fledglings 2012 (*n*)
Abraxas	Male	2002	2010 + 2012	3	2	4	2
Agatha	Female	2011	2012	–	–	0	0
Albright	Female	2006	2010	6	2	–	–
Aleppo	Female	2006	2010 + 2012	5	2	3	3
Ariadne	Female	2008	2012	–	–	3	2
Arion	Female	1999	2010 + 2012	4	2	5	2
Balu	Male	2010	2012	–	–	0	0
Daphne	Female	1999	2010 + 2012	5	2	4	2
Freddy	Male	1999	2010 + 2012	6	0	4	1
Goran	Female	2005	2010 + 2012	5	2	4	2
Heidi	Male	2010	2012	–	–	0	0
Hera	Male	1999	2010	4	2	–	–
Hilda	Male	2009	2012	–	–	0	0
Hombre	Male	2002	2010 + 2012	4	2	5	2
Homer	Female	2006	2010	4	2	–	–
Jarmusch	Male	2005	2012	–	–	4	2
Jule	Male	1998	2010 + 2012	4	2	3	3
Kevin	Male	1997	2010	0	0	–	–
Loki	Female	2006	2012	–	–	4	2
Mammut	Female	2010	2012	–	–	0	0
Manitou	Female	2008	2010	3	0	–	–
Maya	Male	2006	2012	–	–	0	0
Neptun	Female	1999	2010	6	0	–	–
North face	Male	2009	2010 + 2012	0	0	0	0
Ophelia	Female	2011	2012	–	–	0	0
Othello	Female	1999	2010	5	2	–	–
Paco	Male	2010	2012	–	–	0	0
Ritro	Male	2010	2012	–	–	0	0
Rob	Male	2010	2012	–	–	0	0
Salomo	Female	2002	2010	4	1	–	–
Schreckse	Female	2008	2010 + 2012	4	2	4	2
Sesam	-	2009	2010	0	0	–	–
Shannara	Male	2007	2012	–	–	4	2
Simon	Male	2006	2012	–	–	3	2
Skippy	Male	1998	2010	5	2	–	–
Sorrento	Male	2006	2010 + 2012	0	0	4	2
Steppenwolf	Male	2002	2010 + 2012	6	2	4	2
Tiffy	Male	2011	2012	–	–	0	0
Tintifax	Female	2003	2010	3	2	–	–
Waltraut	–	2009	2010	0	0	–	–
Winnetouch	Female	2004	2010 + 2012	4	2	4	2

^a^Sex of two individuals is unknown

### Data collection

Data were collected during two different reproductive seasons: between March and July 2010 (henceforth referred to as Spring 2010) and between February and May 2012 (henceforth referred to as Spring 2012). The aim of the second season of data collection was to integrate the first data set (parasitological examinations, behavioural observations) with additional relevant physiological information (BM, haematological parameters). In both seasons data were collected over a time span of 10 weeks, starting with mating and egg-laying and continuing until feeding and rearing of the young.

#### Weather data

Weather data were provided by a weather station in Gruenau (47°51′E, 13°57′N) operated by Max Rauscher (www.gruenau.tv; last accessed 25 December 2014). Weather data were recorded every 5 min, and daily means were entered into our analysis.

#### Behavioural data

##### *Spring 2010*

During the first 5 weeks of the study, i.e. mating/egg laying phase, the behaviour of the focal birds was monitored during the morning (0800–1000 hours) and afternoon feedings in the aviary (1400–1600 hours) as well as between 1000 hours and 1200 hours in the aviary or on the nearby fields, depending on the location of the focal individuals. Behavioural observations consisted of ten focal observations per individual, with each observation lasting 5 min. The occurrence of all agonistic interactions and the duration of affiliative behaviours were recorded (for a description of the Northern Bald Ibis’s ethogram, see Pegoraro 1992).

##### *Spring 2012*

Behavioural and physiological data were collected daily during the morning feeding of the colony (0800–1100 hours). Consequently, most agonistic interactions were observed in a foraging context. Occurrence of agonistic encounters and the identity of the individuals involved was recorded ad libitum (Martin and Bateson 2007). In total, approximately 115 h of observation were performed over the whole period.

#### Blood samples

Individual blood samples were only collected in February and March 2012 (i.e. Spring 2012) before the start of incubation in order to determine (1) the differential blood cell count (Prinzinger et al. 2012) and (2) HCT. After the morning feedings, focal individuals were caught by hand. The bird’s brachial vein was punctured with a sterile needle (diameter 24 µm), and blood was collected in two heparinized micro-haematocrit capillaries (diameter 75 mm). The procedure lasted <5 min per bird.

To determine an individual’s blood cell count one drop of blood was smeared onto a microscope slide, air-dried and stored until later identification of leucocytes at the University of Veterinary Medicine in Vienna (Austria). Differential blood cell counts provided information on the relative occurrence of different leucocyte types (heterophils, lymphocytes, monocytes, basophils and eosinophils; Prinzinger et al. 2012). Blood smears were stained with a Romanowsky-type stain (Haemaquick; E. Lehmann GmbH, Salzburg Austria) and evaluated microscopically. For the differential blood count, 100 white blood cells were differentiated into heterophilic, eosinophilic or basophilic granulocytes, monocytes, and lymphocytes by oil immersion microscopy at 1000× magnification. The results were given in percentages. The individual arithmetic mean of the two differential counts was calculated for the H/L ratio and used in further statistical analyses. The capillaries were centrifuged at 8000 rpm for 5 min to determine the HCT. Volumes of red blood cells (RBCs) and plasma, respectively, were measured on the capillaries to the nearest 0.5 mm with callipers. HCT was then calculated as a ratio as follow (Sánchez-Guzmán et al. 2004; Prinzinger et al. 2012): RBC volume/(RBC + plasma volume). The individual arithmetic mean of the two HCT values was used in further analyses.

#### Droppings

Individual droppings were collected in regular time intervals over both study periods in order to determine (1) parasite burden (Spring 2010 and 2012) and (2) levels of BM (Spring 2012). Droppings were collected immediately after defecation in individual Eppendorf® microtubes (Eppendorf, Hamburg, Germany) and (1) stored in a refrigerator at +6 °C for analyses within 7 days for parasite burden or (2) stored on ice after collection and frozen at −20 °C within 2 h of sampling for the determination of BM. During both periods of data collection faeces were accurately collected by waiting for a specific individual to defecate in order to be able to attribute the collected faeces exclusively to a given individual and to avoid any cross-contamination. Faecal sample collection was performed independent of behavioural observations.

##### *Spring 2010*

Over the 10 weeks of the study a total number of 466 droppings were collected (mean per focal animal $\bar{\rm x }$ ± SE = 18.6 ± 7.34). Details on the parasitological examination are provided in the "Parasitological examination" section.

##### *Spring 2012*

On average 2.69 ± 1.28 ($\bar{\rm x }$ ± SE) droppings per individual were analysed for parasites and 3.57 ± 0.27 ($\bar{\rm x }$ ± SE) droppings per individual were analysed for BM. Faecal samples are assumed to represent an integrated, proportional record of plasma corticosterone concentration approximately 2–3 h prior defecation, depending on gut passage time (Hirschenhauser et al. 2000; Kotrschal et al. 2000). Analyses were run at the Department for Behavioural Biology, University of Vienna (Austria) where the droppings were assayed by enzyme immunoassay (EIA) according to a method previously validated for geese (Hirschenhauser et al. 2000; Kotrschal et al. 2000; Frigerio et al. 2004) and the Northern Bald Ibis (Sorato and Kotrschal 2006; Dorn et al. 2014). The EIA was performed with functional group-specific antibodies against 11 beta, 21 OH, 20-oxo-corticosterone metabolites. Details about the EIA procedure and cross-reactivities have been published elsewhere (Kotrschal et al. 1998; Frigerio et al. 2004). Concentration limits ranged from 9.87 to 495.70 ng BM/g droppings. Intra- and inter-assay coefficients of variation were determined from homogenized pool samples (10.96 and 4.95 %, respectively). Since birds’ droppings contain both faeces and uric acid crystals, we attempted to collect pure faecal samples, avoiding contamination with uric acid crystals. However, preliminary results indicated that the concentration of steroid metabolites is higher in the solid, faecal fraction than in the uric acid crystals, making the effect of contamination of faeces with urine negligible.

#### Parasitological examination

Examination of faecal samples for excreted parasite products differed between the two periods of data collection. In Spring 2010, a modified flotation technique was applied. First, 0.5 g of the dropping was mixed with 1 ml of saline solution and then centrifuged for 5 min at 2000 rpm. After centrifugation, 1 ml of saline solution was added, and a coverslip was placed on top of the tube so that it touched the top of the meniscus. The coverslip was maintained in this position for 10 min and then carefully removed, with parasite eggs and oocysts being attached to the coverslip (Dryden et al. 2005). In season 2, a flotation method in a McMaster counting chamber was used due to the increased accuracy and sensitivity of this method compared to that used in Spring 2010 (Hiepe et al. 1981). In brief, 0.5–1 g of homogenized faeces was diluted with 5 ml saturated NaCl solution (350 g NaCl, 1000 ml distilled water) and filtered through a sieve to eliminate large food particles and debris. The resulting solution was poured into both McMaster counting chambers where the parasites were counted. A value for eggs/oocysts per gram faeces was calculated according to Hiepe et al. (1981): [number of (coccidian oocysts/nematode eggs) × 5 ml NaCl]/[1 g faeces × 0.30 ml (volume of two counting chambers)].

Each sample was treated as either containing or not containing coccidian oocysts or nematode eggs because (1) two different methods were used to determine the number of parasite eggs and oocysts and (2) there was a low prevalence of parasite eggs and oocysts (Spring 2010: average rate of samples containing nematode eggs $\bar{\rm x }$ ± SE = 0.307 ± 0.087; average rate of samples containing coccidian oocysts $\bar{\rm x }$ ± SE = 0.09 ± 0.021; Spring 2012: average rate of samples containing nematode eggs $\bar{\rm x }$ ± SE = 0.386 ± 0.275; average rate of samples containing coccidian oocysts $\bar{\rm x }$ ± SE = 14.375 ± 2.89). In Northern Bald Ibis several species of coccidia (*Eimeria* sp., *Tyzzeria* spec.), nematodes (Gape worm *Syngamus trachea*, Strongyle worm *Trichostrongylus tenuis* and *Capillaria* spec.) and tapeworms (*Railletina* spec.) were prevalent. This is consistent with the parasite burden found in the population of Greylag geese (*Anser anser*) using the same habitat as the Northern Bald Ibis at the KLF (Wascher et al. 2012) as well as with data collected on other Northern Bald Ibis populations (Mason 2010).

### Statistical Analysis

In Spring 2010, we calculated four zero inflated generalized linear mixed models (ZIGLMM) with binomial error distribution and log link function. Zero inflation was applied to correct for overdispersion resulting from a large number of zero values. Samples containing coccidian oocysts (yes/no; ZIGLMM 1 and 3) and samples containing nematode eggs (yes/no; ZIGLMM 2 and 4) served as response variables. In model 1 and 2 only the first 5 weeks of data collection were analysed. Fixed factors in these models were (1) duration of allopreening received, (2) frequency of aggression won and (3) frequency of aggression lost. The parameters duration of allopreening initiated and sitting in contact with the pair-partner were later omitted from analysis because these factors highly correlated with the factor duration of allopreening received (Spearman’s rho correlations: allopreening initiated–received: *r*_s_ = 0.535, *n* = 222, *p* < 0.001; allopreening received–contact sit: *r*_s_ = 0.463, *n* = 222, *p* < 0.001). The parameters winning and losing aggression were also found to be significantly correlated (Spearman’s rho correlations: *r*_s_ = 0.159, *n* = 222, *p* = 0.018), but as winning or losing an interaction was expected to impact the physiological stress response of an individual in a different way (Wascher et al. 2009) we tested both behavioural parameters. Models 3 and 4 consisted of the entire 10 weeks of data collection, and the fixed factors in these models were week of data taking, temperature, age, sex, pair bond status, number of eggs laid and number of fledglings.

In Spring 2012 we calculated two generalized linear mixed models (GLMMs) with Poisson error distribution and log link function. We used the number of eggs laid (GLMM 5) and number of fledglings (GLMM 6) as response variables. Sex, age, HCT, H/L ratio, mean BM, frequency of aggression won, frequency of aggression lost, mean percentage of samples containing coccidian oocysts and mean percentage of samples containing nematode eggs were included as fixed factors. Subject identities were included as random factors in all ZIGLMMs and GLMMs to control for between-subject variation and unbalanced design. We applied a backwards stepwise elimination procedure and thereby stepwise excluded the least significant factor from the model until only factors with a significance of* p* < 0.1 remained in the final model. All models were calculated in R v. 3.0.2, using the glmmADMB package (version 0.7.7; Skaug et al. 2011).

## Results

In Spring 2012 we found that 55.67 % of the collected samples contained coccidian oocysts. From 22 individuals we collected at least one sample containing coccidian oocysts, whereas from seven individuals none of the collected samples contained coccidian oocysts. In Spring 2010 only 9.68 % of samples contained coccidian oocysts. From 16 individuals we collected at least one faecal sample containing coccidian oocysts, whereas from nine individuals we did not collect a single sample containing coccidian oocysts. In Spring 2012 only 6.19 % of samples contained nematode eggs (*Trichostrongylus tenius:* 5.15 %; unidentified nematode egg: 1.03 %). From 23 individuals we did not collect any samples containing nematode eggs, whereas we did find nematode eggs in samples of six individuals; 1.03 % of the samples of one individual contained tapeworm eggs, whereas in the remaining 28 individuals no tapeworm eggs were detected. In Spring 2010 36.34 % of samples contained nematode eggs (*Syngamus trachea*: 0.43 %; *Ascaridia* spec: 28.17 %; *Capillaria* spec: 0.43 %; *Trichostrongylus tenius*: 0.43 %). From 19 focal individuals we collected at least one sample containing nematode eggs, whereas in six birds we did not collect any faecal samples containing nematode eggs. In Spring 2010 no tapeworm eggs were detected in any faecal samples.

### *Spring 2010*

**Fig. 1:**
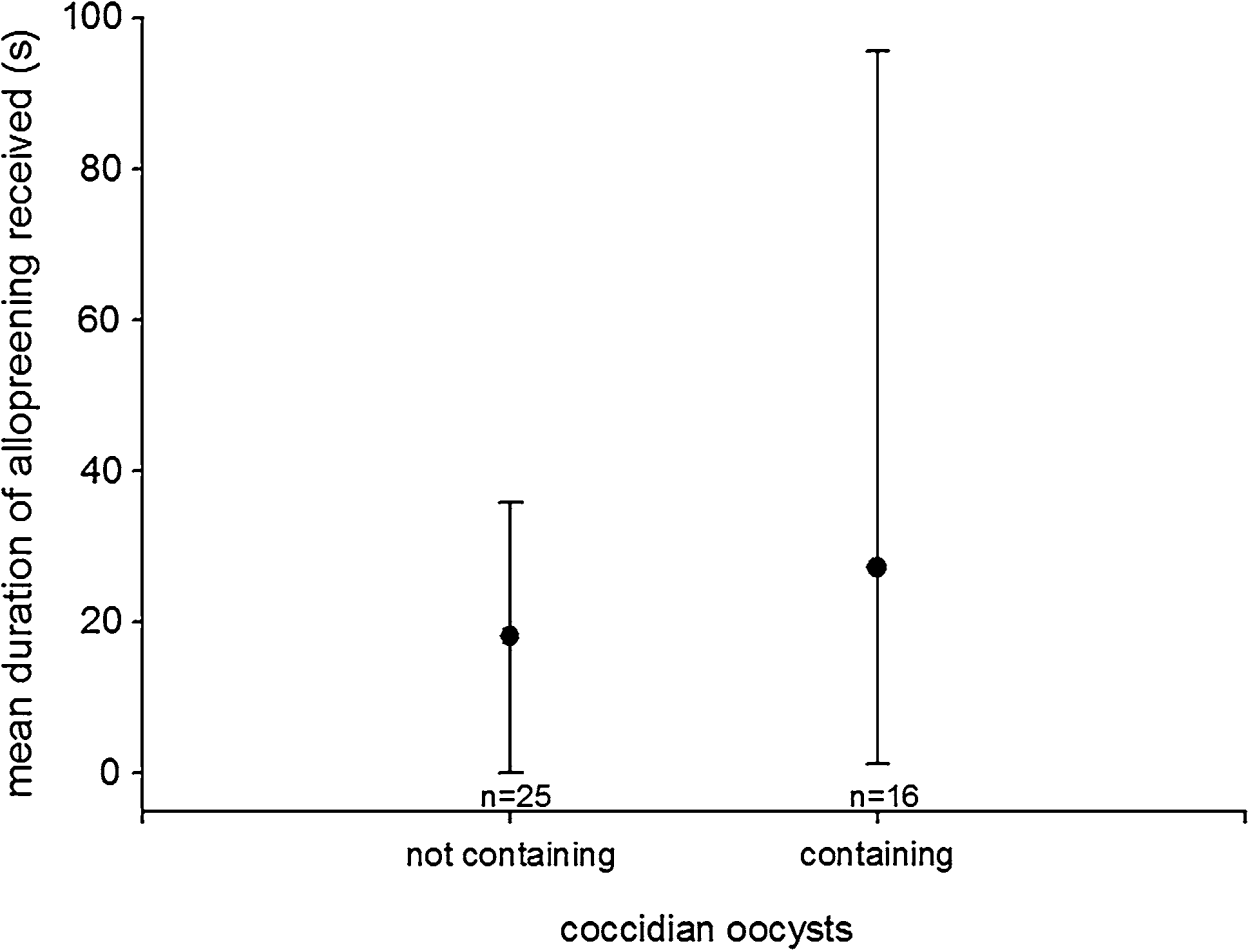
Mean duration of allopreening received in an observation session in relation to coccidian oocyst content of droppings. *Circles* Median, *error bars* interquartile range (25th–75th percentile)

**Fig. 2:**
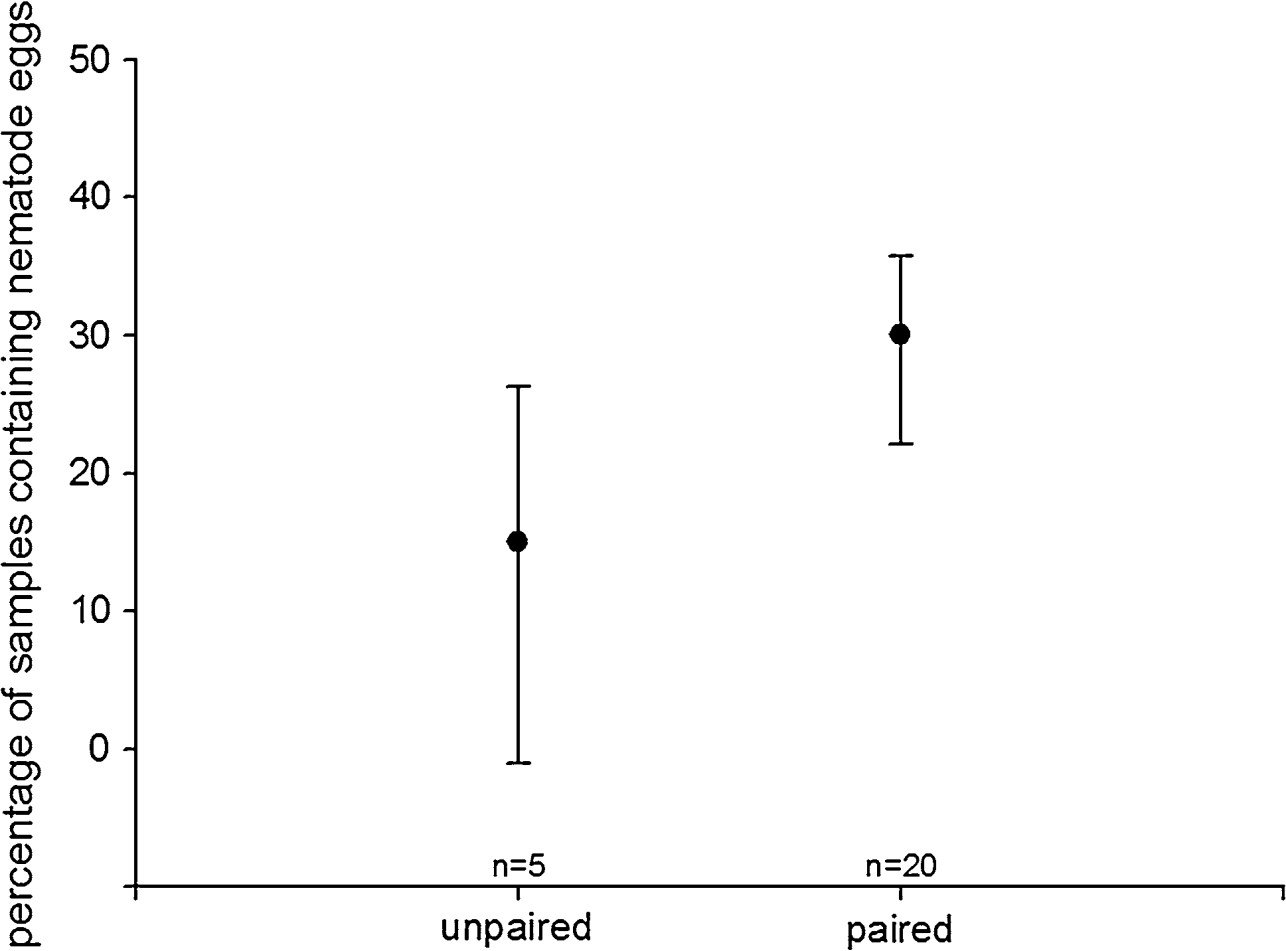
Excretion of nematode eggs depending on pairbond status. *Circles* display median, *error bars* are based on interquartile ranges (lower: 25th and upper: 75th percentile)

Individuals producing a high number of faeces containing coccidian oocysts received more allopreening (ZIGLMM1: estimate ± SE 0.007 ± 0.002, *z* = −2.611, *p* = 0.009; Fig. 1; unless stated otherwise, all data for and related to ZigLMMs and GLMMs in the Results section are given as the estimate ± SE) and won more agonistic interactions than individuals excreting fewer samples containing coccidian oocysts (ZIGLMM1: −0.687 ± 0.301, *z* = −2.281, *p* = 0.022; intercept:  −1.894 ± 0.288, *z* = −6.563, *p* < 0.001). Excretion of samples containing nematode eggs was negatively related to the frequency of aggressive encounters won (ZIGLMM2: −0.63 ± 0.297, *z* = −2.121, *p* = 0.033) and to the frequency of aggressive interactions lost (ZIGLMM2: −1.837 ± 0.492, *z* = −3.733, *p* < 0.001; intercept: 4.554 ± 0.736, *z* = 6.182, *p* < 0.001; Table 2). The amount of droppings containing coccidian oocysts tended to increase with temperature (ZIGLMM3: 0.223 ± 0.114, *z* = 1.959, *p* = 0.05) and increased with the number of eggs an individual incubated (ZIGLMM3: 1.274 ± 0.565, *z* = 2.255, *p* = 0.024) but decreased with the number of offspring fledged (ZIGLMM3: −2.647 ± 1.142, *z* = −2.318, *p* = 0.02; intercept: −2.8 ± 1.335 *z* = −2.096, *p* = 0.036). Excretion of samples containing nematode eggs increased with week of data collection (ZIGLMM4: 0.607 ± 0.147, *z* = 4.122, *p* < 0.001). In general, paired individuals excreted significantly more samples containing nematode eggs than unpaired individuals (ZIGLMM4: 1.820 ± 0.858, *z* = 2.120, *p* = 0.033; Fig. 2). The excretion of samples also containing nematode eggs was significantly higher in females than in males (ZIGLMM4: 1.837 ± 0.435, *z* = 4.220, *p* < 0.001).

### *Spring 2012*

Number of eggs laid (GLMM5: 0.148 ± 0.029, *z* = 5.017, *p* < 0.001) and fledged young (GLMM6: 0.14 ± 0.039, *z* = 3.52, *p* < 0.001; intercept:  −0.801 ± 0.371, *z* = −2.157, *p* = 0.03) increased significantly with age. In addition, number of eggs laid tended to increase with the percentage of samples containing coccidian oocysts (GLMM6: 0.006 ± 0.003, *z* = 1.848, *p* = 0.064; intercept: −0.006 ± 0.345, *z* = −1.739, *p* = 0.082). No significant relationship between parasite burden, any physiological parameters (HCT, H/L ratio, BM), age or behaviour was detected (Table 2).

Full statistical models are presented in Table 2.

**Table 2:** Results of the full statistical models

Full statistical models	Estimate ± standard error	*z*	*p*
ZIGLMM 1			
**(Intercept)**	−**1**.**894** ± **0**.**288**	−**6**.**563**	<**0**.**001**
**Allopreening received**	**0**.**007** ± **0**.**002**	**2**.**611**	**0**.**009**
Aggression won	−0.06 ± 0.12	−0.505	0.612
**Aggression lost**	−**0**.**687** ± **0**.**301**	−**2**.**28**	**0**.**022**
ZIGLMM 2			
(Intercept)	4.554 ± 0.073	6.182	<0.001
Allopreening received	0 ± 0.003	0.207	0.835
**Aggression won**	−**0**.**63** ± **0**.**297**	−**2**.**121**	**0**.**033**
**Aggression lost**	−**1**.**837** ± **0**.**492**	−**3**.**733**	<**0**.**001**
ZIGLMM 3			
**(Intercept)**	−**4**.**459** ± **3**.**289**	−**1**.**355**	**0**.**175**
Week	0.120 ± 0.546	0.220	0.825
**Temperature**	**0**.**210** ± **0**.**152**	**1**.**377**	**0**.**168**
Age	0.096 ± 0.230	0.416	0.677
**Sex**	**1**.**574** ± **1**.**568**	**1**.**003**	**0**.**315**
Pairbond status	4.967 ± 5.253	0.945	0.344
**Number of eggs**	**0**.**754** ± **0**.**623**	**1**.**210**	**0**.**226**
**Number of fledglings**	−**3**.**822** ± **2**.**600**	−**1**.**469**	**0**.**141**
ZIGLMM 4			
**(Intercept)**	−**5**.**550** ± **1**.**224**	−**4**.**532**	<**0**.**001**
**Week**	**0**.**564** ± **0**.**209**	**2**.**692**	**0**.**007**
Temperature	0.020 ± 0.053	0.386	0.699
Age	−0.084 ± 0.065	−1.292	0.196
**Sex**	**1**.**536** ± **0**.**500**	**3**.**069**	**0**.**002**
**Pairbond status**	**1**.**792** ± **1**.**374**	**1**.**304**	**0**.**192**
Number of eggs	0.045 ± 0.211	0.214	0.830
Number of fledglings	−0.020 ± 0.273	−0.075	0.939
GLMM 5			
(Intercept)	2.238 ± 1.854	1.207	0.227
Sex	0.01 ± 0.344	0.031	0.974
**Age**	**0**.**152** ± **0**.**042**	**3**.**556**	<**0**.**001**
HCT	−5.413 ± 3.945	−1.371	0.17
HL ratio	0.006 ± 0.077	0.085	0.932
BM	0.003 ± 0.003	1.052	0.292
Aggression won	−0.016 ± 0.024	−0.677	0.498
Aggression lost	−0.051 ± 0.039	−1.285	0.198
**% of samples containing coccidian oocysts**	**0**.**004** ± **0**.**004**	**0**.**955**	**0**.**339**
% of samples containing nematode eggs	−0.006 ± 0.007	−0.932	0.35
GLMM 6			
(Intercept)	1.139 ± 2.569	0.443	0.657
Sex	0.209 ± 0.471	0.444	0.656
Age	**0**.**132** ± **0**.**058**	**2**.**251**	**0**.**024**
HCT	−5.663 ± 5.4	−0.863	0.387
HL ratio	−0.021 ± 0.108	−0.2	0.841
BM	0.006 ± 0.004	1.341	0.179
Aggression won	−0.02 ± 0.034	−0.599	0.549
Aggression lost	−0.056 ± 0.055	−1.014	0.31
% of samples containing coccidian oocysts	0.000 ± 0.006	0.157	0.875
% of samples containing nematode eggs	−0.013 ± 0.011	−1.216	0.223

Factors in bold remained in the final model

## Discussion

The results of our study demonstrate correlations between social behaviour, excreted immune-reactive corticosterone metabolites (BM), haematology parameters, parasite excretion patterns and reproductive output in the Northern Bald Ibis. As expected, social behaviour and excreted parasite eggs and oocysts were related to each other. Individuals receiving much allopreening and sitting close to their partners showed high excretion rate of coccidian oocysts. Similar evidence can be found in the literature, where individuals exchanging high rates of preening are also more likely to transmit parasites to one another. Recent results from a study on a wild group of Japanese macaques (*Macaca fuscata yakui*; MacIntosch et al. 2012) suggest that grooming networks mediate exposure to certain nematode species. It is also generally known that social contacts may facilitate the transmission of ectoparasites, whereas the transmission of endoparasites happens primarily through the ingestion of faeces (e.g. Hoogland and Sherman 1976; Arnold and Lichtenstein 1993; Johnson et al. 2004). The effect of group size on endo- and ectoparasite load is still open to discussion, with conflicting results from such studies. In Cape ground squirrels, a highly social rodent, parasite load was found not to be related to group size (*Xerus inauris*, Hillegass et al. 2008), whereas in birds a seasonal dependent positive relationship between levels of parasitism and colony size has been shown (e.g. Brown and Brown 1986; Fecchio et al. 2011).

An alternative explanation is that a high allopreening rate may reflect the response of the pair-partner to a social need and that it serves as an emotional link to behaviour and physiology (Aureli and Smucny 2000). Affiliative behaviours, such as allopreening/grooming, and the presence of social allies may actually buffer the negative effects of social stress (e.g. Creel 2001; Mendl 2001; Sgoifo et al. 2001; mammals: Boccia et al. 1989; Levine 1993; Sachser et al. 1998; Hennessy et al. 2006; birds: Frigerio et al. 2003; Scheiber et al. 2005; Stöwe et al. 2008). In our study individuals receiving much allopreening also showed low levels of agonistic interactions, suggesting that allopreening among the Northern Bald Ibis may also be interpreted as an investment in emotional and social support of a partner towards containing the detrimental effect of high parasite load (Hillegass et al. 2010).

Paired individuals excreted significantly more nematode eggs than unpaired individuals. This may be related to stress levels associated with social status and with seasonal changes in reproduction investment (e.g. Creel et al. 1996; Kotrschal et al. 1998). A central hypothesis of ecological immunology is that immune defence is traded off against competing physiological and behavioural processes, which is expected to be most pronounced during energetically demanding periods, such as reproduction (Norris and Evans 2000). In the Great tit (*Parus major*), reproductive effort was shown to increase susceptibility to haematozoan infections in females (Norris et al. 1994). In the long-lived Common eider (*Somateria mollissima*) immune function was found to be reduced in females incubating large clutches (Hanssen et al. 2005). We suggest that the high parasite load in the paired Northern Bald Ibis individuals in our study is related to a time constraint resulting from the high energetic demands during reproduction, which may in turn affect the immune system. In our study, females generally excreted more nematode eggs than males. This observation is in line with previous results on Greylag geese where females were found to excrete more coccidian oocysts than males (Wascher et al. 2012). Greylag geese females incubate the eggs and brood the goslings and therefore also after egg production bear a greater reproductive investment than males, which are mainly active in vigilance and in the defence of their family against other geese (e.g. Lazarus and Inglis 1986; Schindler and Lamprecht 1987; Black and Owen 1989; Williams et al. 1994). In contrast, males and females of the Norther Bald Ibis share relatively equally most of the parental duties (e.g. incubation, chick feeding), with the exception of egg-laying (Pegoraro 1992; Böhm and Pegoraro 2011). However, egg-laying is known to be energetically costly and a limiting factor for parental fitness (Monaghan and Nager 1997). Therefore, the described higher parasite load in females compared to males could be a result of a decreased immune system in response to the high energetic demands of egg-laying.

Northern Bald Ibis males and females are relatively symmetrical in terms of androgen levels (Sorato and Kotrschal 2006). Androgens vary according to the specific breeding system (Hirschenhauser et al. 2003) and have previously been described to have an immunosuppressing effect, especially in males (immunocompetence handicap hypothesis: Folstad and Karter 1992; Saino et al. 1995; Ros 1999; Wingfield et al. 1999). However, a recent meta-analysis found no effect of testosterone on direct measures of immunity, suggesting that the described immunosuppressive effects of testosterone may be due to its linkage with corticosterone (Roberts et al. 2004).

We also found that parasite burden was positively correlated with the number of eggs laid, but negatively correlated with the number of fledglings. This may be linked with the seasonal temperature increase, which may favour the spread of parasites. Alternatively, this result is in agreement with the findings of other correlative studies showing that poorly reproducing individuals also have high parasite loads (e.g. Möller 1990; Richner et al. 1993). Since parasite levels were measured before and during incubation as well as during chick-rearing, we suggest that the high investments in egg production constrain caregiving towards chicks by the parasitized female, leading to a reduced number of fledglings by the parasitized female. In fact, experimental manipulation of parental effort or brood size showed that birds forced to work had a decreased ability to respond to an immune system challenge (Nordling et al. 1998; Deerenberg 1996; Deerenberg et al. 1997) and, for example, were susceptible to avian malaria (Nordling et al. 1998; Richner et al. 1995). This may, in turn, constrained fledging success. In House martins (*Delichon urbica*), infection with blood parasites had a negative effect on clutch size, hatching and fledging success (Marzal et al. 2005). Other studies (e.g. Richner et al. 1995) have suggested that the interaction between parasite infection, nutrition and reproductive effort could be an important mechanism in shaping life-history variation in avian populations.

We found no significant correlation between BM and any of the haematological parameters or with parasite burden. Increased parasite excretion may affect baseline glucocorticoid levels (Mougeot et al. 2010) and reflect shifts in leukocyte composition, such as an increase in heterophils and a decrease in lymphocytes. For this reason, the H/L ratio is generally used as an indicator of physiological stress (e.g. Vleck et al. 2000; Davis et al. 2008). However, similar results were found in an experimental situation in Greylag geese (S. Ludwig, unpublished data), suggesting a buffering effects of conspecifics on an individual’s stress response. It should be noted here that we cannot exclude the possibility that the characteristics of our sampling method account for the lack of a relationship between BM and parasite burden.

In conclusion, the results of our study suggest that social behaviour, parasite burden and physiology are linked in a complex manner, which may be a principle not only in birds but in social vertebrates in general. In the context of conservation physiology (Stevenson et al. 2005; Wikelski and Cooke 2006), our results also contribute background information which may support Northern Bald Ibis re-introduction and conservation programmes (e.g. Dutton et al. 2002; Sánchez-Guzmán et al. 2004; Villegas et al. 2004).
